# Light tolerance in light-tolerant photosynthetic organisms: a knowledge gap

**DOI:** 10.1093/jxb/erae338

**Published:** 2024-08-05

**Authors:** Guy Levin, Gadi Schuster

**Affiliations:** Faculty of Biology, Technion, Haifa, 32000, Israel; Faculty of Biology, Technion, Haifa, 32000, Israel; Grand Technion Energy Program, Technion, Haifa, 32000, Israel; Bielefeld University, Germany

**Keywords:** *Chlorella ohadii*, crop yield, improving photosynthesis, light stress, light tolerance, photoinhibition, photoprotection, photosynthesis

**The absorption of excess light may lead to the inhibition of photosynthesis in a process known as photoinhibition. Understanding the mechanisms of protection from photoinhibition is key for research aiming to enhance crop productivity by generating resilient plants. Substantial insights into photoprotection and high-light tolerance have been gained from model organisms including Arabidopsis and unicellular algae such as *Chlamydomonas* and *Chlorella* species. However, these organisms are not necessarily high-light tolerant and so are not ideal candidates for investigating photoprotection. Here, we highlight the importance of researching photoprotection in high-light tolerant organisms, which are expected to harbor the most efficient protective mechanisms.**

In some cases photoinhibition does not impair growth but rather increases biomass (Close and Beadle, 2003; Adams *et al.*, 2013). However, when the rate of light-induced oxidative damage to the photosynthetic proteins exceeds the rate of repair, photoinhibition may result in bleaching and compromised growth. Improving photosynthesis in crops to enhance their yield has been a long-standing goal for many scientists (Long *et al.*, 2006; Zhu *et al.*, 2010; Leister, 2023; Wu *et al.*, 2023; Croce *et al.*, 2024). Several approaches have been proposed, including enhancing carbon fixation, enhancing photosynthetic activity, and improving photoprotection-related mechanisms. A breakthrough was made in 2016 when tobacco plants were transformed to express Arabidopsis PsbS, violaxanthin de-epoxidase, and zeaxanthin epoxidase, three major factors in photoprotection. PsbS induces non-photochemical quenching (NPQ) mechanisms in plants, in which light energy is dissipated as heat rather than being used for photosynthesis, thus protecting the organism from high-light (HL) intensities. Violaxanthin de-epoxidase promotes NPQ by transforming violaxanthin to zeaxanthin under HL stress in a process known as the xanthophyll cycle. Excess energy stored in chlorophylls is transferred to zeaxanthin, where it is dissipated. Zeaxanthin epoxidase catalyses the transformation of zeaxanthin to violaxanthin when HL stress is alleviated, fostering the change from a photoprotective to a photosynthetic mode, where harvested light promotes growth. The field-grown transgenic tobacco plants produced 15% more biomass compared with wild-type plants, an improvement that was attributed to a faster recovery from NPQ upon relaxation from HL while maintaining maximal photoprotective capabilities under HL stress (Kromdijk *et al.*, 2016). Application of the same method in soybeans resulted in up to 33% increase in seed yield (De Souza *et al.*, 2022). In another study, overexpression of PsbS in field-grown tobacco led to a decrease in stomatal opening, resulting in a 25% reduction in water loss per assimilated CO_2_ (Głowacka *et al.*, 2018). These reports highlight the potential of exploiting known photoprotective proteins to improve photoprotection-related mechanisms and enhance crop growth, yield, and water-use efficiency. But can they be improved even further?

## Understanding photoprotection requires the study of the most protected organisms

**Fig. 1.**
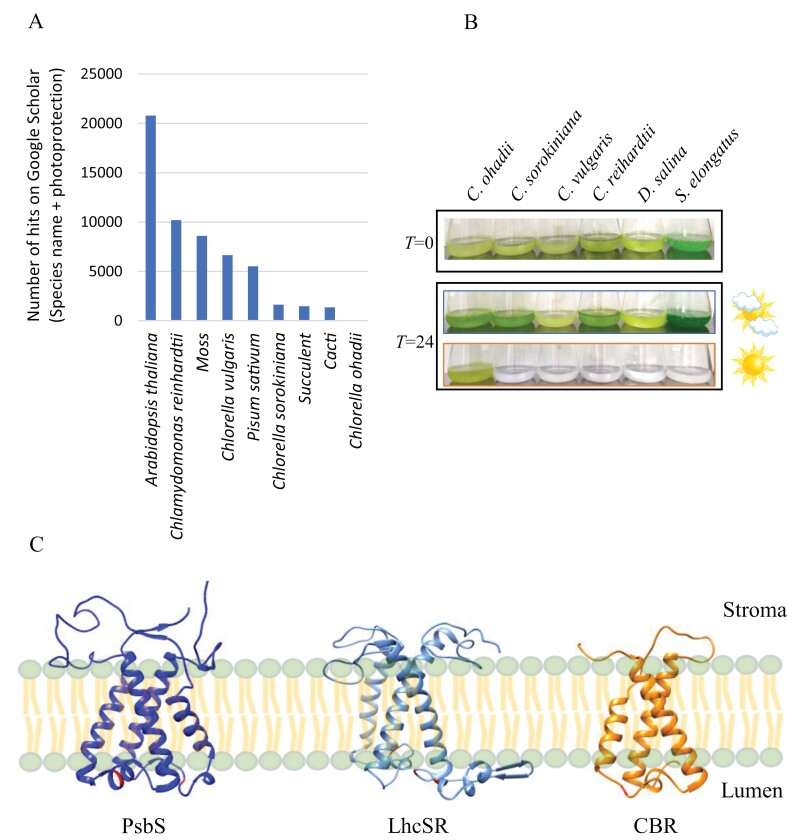
Photoprotection research is mainly performed in non-light-tolerant organisms. (A) Number of hits on Google Scholar search for the keywords ‘photoprotection’ + the indicated organism. (B) *Chlorella ohadii* thrives at light intensities that are fatal to most model algae. Organisms were grown for 24 h at low or high light intensities (100 and 2500 μmol photons m^–2^ s^–1^). *T* is hours since the transition to low or high conditions. Panel adapted from Levin *et al.* (2023). (C) *Chlorella ohadii* lacks the non-photochemical quenching-inducing proteins PsbS and LhcSR. Instead, it accumulates massive quantities of CBR, a protein with a similar structure but unknown function, under high-light growth conditions. The structure of PsbS was imported from PDB (ID: 4RI3). Models of the CBR of *C. ohadii* and LhcSR of *Chlamysdomonas reinhardtii* were created using AlphaFold. Proteins are presented from the side of the thylakoid membrane. Panel adapted from Levin and Schuster (2023). CBR, carotenoid biosynthesis-related; LhcSR, light-harvesting complex stress-related.

Understanding the molecular mechanisms underlying photoprotection is key to improving photosynthesis in crops. Improving photoprotection will expectedly improve crop growth under HL conditions by minimizing the detrimental effects of excess light and enhancing tolerance to HL. While highly light-tolerant organisms are seemingly ideal candidates for studying photoprotection, the majority of research in this field has been performed using model organisms including the vascular plant Arabidopsis and other plants or the green microalgae *Chlamydomonas reinhardtii* and various *Chlorella* species (Fig. 1A) (Tian *et al.*, 2017). This choice may be due to the established protocols and resources that have accumulated over dozens of years of research, as well as developing the appropriate genetic tools and mutant collections. Yet, it is important to note that model organisms are often not specifically tolerant to HL. This poses two major limitations to photoprotection research. First, as these organisms are mostly not perfectly adapted to HL conditions, they are likely not equipped with the most efficient photoprotective mechanisms. Secondly, these organisms cannot always maintain regular growth rates under HL intensities, limiting the study of photoprotection under extreme HL levels. For instance, Arabidopsis and *Chlamydomonas reinhardtii* ideally grow under light intensities of approximately 50–150 µmol photons m^–2^ s^–1^, although HL-tolerant *Chlamydomonas* mutants that maintain high growth rates have been reported (Förster *et al.*, 2005) and some Arabidopsis species are more HL-tolerant compared with others. HL-tolerant photosynthetic organisms, such as cacti, are ideally grown under much higher light intensities where the photoprotection mechanisms that were discovered in model organisms may not be sufficient. This tolerance may be governed by unknown photoprotective mechanisms that are highly efficient and enable these organisms to withstand extreme light intensities. Crucially, they are expected to be different from those of commonly researched model organisms due to prolonged evolution under constant extreme HL stress. While in some cases enhancement of the activity or slight modification of known photoprotective mechanisms proved sufficient for enhancing crop yield or water user efficiency (Kromdijk *et al.*, 2016; Głowacka *et al.*, 2018; De Souza *et al.*, 2022), it is expected that utilizing the most efficient photoprotective mechanisms from extremely HL-tolerant organisms could further benefit the plant.

## The case of *Chlorella ohadii*

Recently, we reported that *Chlorella ohadii*, an extremely HL-tolerant green microalga that thrives in the desert, can maintain maximal growth rates in light intensities that have proven fatal to other tested photosynthetic organisms (Fig. 1B). Its photoinhibition protection mechanisms are uniquely different from those described in typical model organisms (Levin *et al.*, 2021, 2023, 2024). Interestingly, we found that *C. ohadii* lacks the highly important known photoprotection components of model plants and algae. Notably, under HL growth conditions (2000 µmol photons m^–2^ s^–1^), *C. ohadii* does not accumulate PsbS and its algal homolog, the light-harvesting complex stress-related (LhcSR) protein, which drives non-destructive dissipation of excess energy through NPQ. Additionally, state transitions, in which light-harvesting complexes of photosystem II (LHCII) are transferred to photosystem I to reduce excitation pressure on the sensitive photosystem II, do not occur in HL-grown *C. ohadii* (Levin *et al.*, 2023). Instead, HL-grown *C. ohadii* eliminates LHCII, leaving an antenna-less PSII core that absorbs much less light energy, which is expected to massively reduce the generation of toxic reactive oxygen species. Moreover, carotenoid biosynthesis-related (CBR), an LHC-like protein with an unknown function and a similar structure to PsbS and LhcSR, is extensively accumulated in HL-grown *C. ohadii* (Fig. 1C) (Levin and Schuster, 2023; Levin *et al.*, 2023). CBR was previously suggested to bind carotenoids (Levy *et al.*, 1993), and in our work, co-localized with high quantities of zeaxanthin and lutein, two carotenoids of great importance to photoprotection (Levin *et al.*, 2023). Counterintuitively, HL-grown *C. ohadii* accumulates fewer PSI degradation products compared with low-light grown cells, suggesting that the photosynthetic proteins are better protected under HL growth conditions (Levin *et al.*, 2024). This unique photoprotective mechanism, which is distinct from those of other model algae and plants, has proven to be more efficient, as highlighted by the capabilityof *C. ohadii* to thrive under higher light intensities (Fig. 1B) (Levin *et al.*, 2023).

## Identifying novel targets for improving photoprotection from the most specialized organisms

Little is known about the photoprotection mechanisms of specifically HL-tolerant photosynthetic organisms, as research is largely focused on a few select model species (Fig. 1A). Despite the limited expertise with specialized models, efforts should be invested to expand research to include them, as they are likely to feature novel and unique mechanisms, as in the case of *C. ohadii*. From a practical perspective, studying photoprotection in HL-tolerant organisms will uncover novel targets and be highly beneficial for improving photosynthesis in crops. As mentioned above, transforming crops to express photoprotective proteins enhanced their growth, yield, and water-use efficiency. However, it is important to note that using similar methods in Arabidopsis and potatoes did not improve yields (Garcia-Molina and Leister, 2020; Lehretz *et al.*, 2022). We suggest that outcomes can be improved by introducing photoprotective mechanisms from specialized HL-tolerant organisms to crops grown under constant HL. For instance, our work flagged CBR, which is structurally similar to PsbS and likely plays a key role in the photoprotection of *C. ohadii*, as an obvious target for enhancing photoprotection (Fig. 1C) (Levin and Schuster, 2023; Levin *et al.*, 2023). However, a better understanding of the CBR-dependent photoprotection mechanism is required for informed integration into the photoprotection landscape of important crops. We also identified a significant accumulation of omega-3 fatty acid desaturase in HL-grown cells (Levin *et al.*, 2021). The enzyme synthesizes polyunsaturated fatty acids, which can act as sinkholes for harmful reactive oxygen species that are produced under HL conditions, thereby protecting the photosynthetic proteins. Moreover, the elimination of the LHCII in *C. ohadii* significantly reduces the amount of absorbed excess energy in the first place, minimizing photo-induced damage. In many algal species, truncated antennae are associated with enhanced biomass production (Cazzaniga *et al.*, 2014; Jeong *et al.*, 2017). In plants, models suggested that a 60% reduction in the chlorophyll content can lead to a 14% rise in nitrogen efficiency and a modest 3% increase in canopy photosynthesis. Furthermore, it is predicted that ideally allocating the resulting conserved nitrogen to other photosynthetic components may increase both nitrogen efficiency and canopy photosynthesis by more than 30% (Song *et al.*, 2017).

## Future research

Improving photosynthesis and photoprotection-related mechanisms has proven to be a viable approach to increasing crop yields. However, the photoprotective components currently used to enhance photoprotection mostly originate from non-HL-tolerant model organisms. We suggest expanding the pool of organisms we use for photoprotection research to include more organisms that are highly tolerant to extreme light intensities. This will enable us to identify novel, highly specialized targets to improve photoprotection and photosynthesis in important crops, further increasing their growth and yield. Moreover, we suggest that revisiting older HL stress and acclimation studies with the current molecular and mechanistic understanding would be highly informative to deepen our understanding of photoinhibition and photoprotection mechanisms.
